# Supplementary material for: The impact of companies disclosing ESG reports in multiple languages on the enthusiasm of foreign investors for holding shares
Source: PLoS One. 2024 Mar 7;19(3):e0299643. doi: 10.1371/journal.pone.0299643 (PMC10919622; doi:10.1371/journal.pone.0299643)
Supplement: S1 Appendix — (DOCX) [file pone.0299643.s001.docx]

**Appendix**

**Statistics on disclosure and verification of ESG reports, 2006-2022**

| **Year** | **Number of listed enterprises** | **The number of ESG reports disclosed by companies** | **Percentage of companies that disclose ESG reports** | **Number of ESG confirmations** | **The proportion of ESG report assurance to the number of ESG reports disclosed by enterprises** |
| --- | --- | --- | --- | --- | --- |
| 2006 | 1434 | 1340 | 93.44% | 0 | 0.00% |
| 2007 | 1550 | 1460 | 94.19% | 0 | 0.00% |
| 2008 | 1625 | 1513 | 93.11% | 11 | 0.73% |
| 2009 | 1718 | 1657 | 96.45% | 10 | 0.60% |
| 2010 | 2063 | 2002 | 97.04% | 19 | 0.95% |
| 2011 | 2342 | 2227 | 95.09% | 22 | 0.99% |
| 2012 | 2472 | 2345 | 94.86% | 38 | 1.62% |
| 2013 | 2489 | 2390 | 96.02% | 34 | 1.41% |
| 2014 | 2587 | 2500 | 96.64% | 33 | 1.31% |
| 2015 | 2806 | 2688 | 95.79% | 30 | 1.11% |
| 2016 | 3052 | 2977 | 97.54% | 38 | 1.26% |
| 2017 | 3440 | 3353 | 97.47% | 55 | 1.62% |
| 2018 | 3567 | 3454 | 96.83% | 48 | 1.37% |
| 2019 | 3777 | 3659 | 96.88% | 44 | 1.18% |
| 2020 | 4140 | 4106 | 99.18% | 82 | 1.94% |
| 2021 | 4669 | 4628 | 99.12% | 127 | 2.62% |
| 2022 | 5157 | 1732 | 33.59% | 48 | 2.77% |

The disclosure rate of listed companies’ ESG reports also reached more than 90%. The rate of ESG reports assurance is less than 2% by 2020, and the rate of ESG reports assurance is 2% or more by 2021-2022.
