# Supplementary material for: The impact of companies disclosing ESG reports in multiple languages on the enthusiasm of foreign investors for holding shares
Source: PLoS One. 2024 Mar 7;19(3):e0299643. doi: 10.1371/journal.pone.0299643 (PMC10919622; doi:10.1371/journal.pone.0299643)
Supplement: Attachment — Submitted filename: Response to Reviewers.docx [file pone.0299643.s003.docx]

Dear editor,

Our most sincere thanks to you, Reviewer 3 for the very thoughtful review and helpful comments, and suggestions on our writing in this round.

We provide the original comments from Reviewer 3 in boldface and our responses to each item in regular font. We highlight the main amendment in blue in the revised manuscript.

**Response to Reviewer 3**

（PONE-D-23-30913R1）

**1.There are still some corrections to be made within the text concerning minor typos and apparently errors for references.**

Thanks for your helpful suggestion.

According to the reviewer’s comments, we have revised the manuscript’s format, punctuation and typos. There are some grammatical errors in the sentences, and we have corrected them. In the manuscript, we have highlighted the revisions in the text in blue font.

The references corrected in the manuscript are highlighted in blue. The collated references are listed as follows:

1. Eleonora,B.Capital Structure and Innovation:Causality and Determinants.Empirical .2013;40(1),111-151.https://doi.org/10.1007/s10663-011-9179-y.
2. Hain, D., Johan, S., Wang, D. Determinants of Cross-Border Venture Capital Investments in Emerging and Developed Economies: The Effects of Relational and Institutional Trust. Journal of Business Ethics.2016;138:743–764. https://doi.org/10.1007/s10551-015-2772-4.
3. Chen H,Chen J Z,Lobo G J,et al.Effect s of audit quality on earnings management and cost of equity capital:Evidence from China.Contemporary Accounting Research.2011; 28(3):892-925.<https://doi.org/10.1111/j.1911-3846.2011.01088.x>.
4. An, J., Jiang, M., Xu, J.Professional norms and risk-taking of bank employees: Do expectations of peers’ risk preferences matter?. Journal of Financial Stability.2021; 56(C):1-13.[https://doi.org/10.1016/j.jfs.2021.100938](https://doi.org/10.1016/j.jfs.2021.100938).
5. So Ra Park, Jae Young Jang.The lmpact of ESG Management on Investment Decision: InstitutionalInvestors’ Perceptions of Country-Specific ESG Criteria.International Journal Financial Studies. 2021; 9(3): 1-27.https://doi.org/10.3390/ijfs9030048
6. llze Zumente, Natalja Lace.ESG Rating Necessity for the Investor or the Company?.Sustainability. 2021;13(16):1-14. https://doi.org/10.3390/su13168940.
7. [Bao Wu](https://www.researchgate.net/profile/Bao-Wu-3?_tp=eyJjb250ZXh0Ijp7ImZpcnN0UGFnZSI6InB1YmxpY2F0aW9uIiwicGFnZSI6InB1YmxpY2F0aW9uIn19),[Qiuyang Gu](https://www.researchgate.net/scientific-contributions/Qiuyang-Gu-2227564037?_tp=eyJjb250ZXh0Ijp7ImZpcnN0UGFnZSI6InB1YmxpY2F0aW9uIiwicGFnZSI6InB1YmxpY2F0aW9uIn19),[Zijia Liu](https://www.researchgate.net/scientific-contributions/Zijia-Liu-2239291096?_tp=eyJjb250ZXh0Ijp7ImZpcnN0UGFnZSI6InB1YmxpY2F0aW9uIiwicGFnZSI6InB1YmxpY2F0aW9uIn19),[Jiaqiang Liu](https://www.researchgate.net/scientific-contributions/Jiaqiang-Liu-2256939441?_tp=eyJjb250ZXh0Ijp7ImZpcnN0UGFnZSI6InB1YmxpY2F0aW9uIiwicGFnZSI6InB1YmxpY2F0aW9uIn19).Clustered Institutional Investors, Shared ESG Preferences, and Low-carbon Innovation in Family Firms.[Technological Forecasting and Social Change](https://www.researchgate.net/journal/Technological-Forecasting-and-Social-Change-0040-1625?_tp=eyJjb250ZXh0Ijp7ImZpcnN0UGFnZSI6InB1YmxpY2F0aW9uIiwicGFnZSI6InB1YmxpY2F0aW9uIn19).2023;194(1):1-25.https://doi.org/[10.1016/j.techfore.2023.122676](http://dx.doi.org/10.1016/j.techfore.2023.122676).
8. Hatem Riiba, Samir Saadi, Sabri Boubaker, Sara Xiaova Ding.Annual report readability andthe cost of equity capital.Journal of Corporate Finance.2021;67(C):1-25.https://doi.org/101902.10.1016/j.jcorpfin.2021.101902.

**2.Please check if the numbers that appear within the text should be linked to references in your reference list.**

Thanks for your helpful suggestion.

After carefully reading the reviewers’ comments and manuscripts, we adopted footnotes to better explain the source of the data in the text.

As a footnote, we explain the numbers mentioned within the text, namely that “less than 2% of listed Chinese companies have ESG reporting checks” and “China has become the world’s largest FDI outflow, with a total investment of $133 billion.”

The numbers mentioned in “less than 2% of listed companies in China have conducted ESG report verification” is our manual statistical collation based on ESG verification reports issued by China’s Shenzhen Stock Exchange and Shanghai Stock Exchange from 2006 to 2022. Our statistical results are listed in the appendix.

“China has become the world's largest FDI outflow with a total investment of US $133 billion”, according to the World Investment Report 2021, available on the UNCTAD website (https://unctad.org/).

**Appendix**

**Statistics on disclosure and assurance of ESG reports, 2006-2022**

| **Year** | **Number of listed enterprises** | **The number of ESG reports disclosed by companies** | **Percentage of companies that disclose ESG reports** | **Number of ESG confirmations** | **The proportion of ESG report assurance to the number of ESG reports disclosed by enterprises** |
| --- | --- | --- | --- | --- | --- |
| 2006 | 1434 | 1340 | 93.44% | 0 | 0.00% |
| 2007 | 1550 | 1460 | 94.19% | 0 | 0.00% |
| 2008 | 1625 | 1513 | 93.11% | 11 | 0.73% |
| 2009 | 1718 | 1657 | 96.45% | 10 | 0.60% |
| 2010 | 2063 | 2002 | 97.04% | 19 | 0.95% |
| 2011 | 2342 | 2227 | 95.09% | 22 | 0.99% |
| 2012 | 2472 | 2345 | 94.86% | 38 | 1.62% |
| 2013 | 2489 | 2390 | 96.02% | 34 | 1.41% |
| 2014 | 2587 | 2500 | 96.64% | 33 | 1.31% |
| 2015 | 2806 | 2688 | 95.79% | 30 | 1.11% |
| 2016 | 3052 | 2977 | 97.54% | 38 | 1.26% |
| 2017 | 3440 | 3353 | 97.47% | 55 | 1.62% |
| 2018 | 3567 | 3454 | 96.83% | 48 | 1.37% |
| 2019 | 3777 | 3659 | 96.88% | 44 | 1.18% |
| 2020 | 4140 | 4106 | 99.18% | 82 | 1.94% |
| 2021 | 4669 | 4628 | 99.12% | 127 | 2.62% |
| 2022 | 5157 | 1732 | 33.59% | 48 | 2.77% |

The disclosure rate of listed companies’ ESG reports also reached more than 90%. The rate of ESG reports assurance is less than 2% by 2020, and the rate of ESG reports assurance is 2% or more by 2021-2022.
