# Supplementary material for: The impact of companies disclosing ESG reports in multiple languages on the enthusiasm of foreign investors for holding shares
Source: PLoS One. 2024 Mar 7;19(3):e0299643. doi: 10.1371/journal.pone.0299643 (PMC10919622; doi:10.1371/journal.pone.0299643)
Supplement: Attachment — Submitted filename: Response to Reviewers.docx [file pone.0299643.s004.docx]

Dear editor,

Our most sincere thanks to you, editor for the very thoughtful review and helpful comments, and suggestions on our writing in this round.

We provide the original comments from editor in boldface and our responses to each item in regular font. We highlight the main amendment in blue in the revised manuscript.

**Response to Editor**

（PONE-D-23-30913R2）

**1.For example, the last paragraph before the Literature review reads: “One type of literature is the study of the effects of the introduction of different types of equity on corporate governance and corporate performance 10. Another type of literature is the study of factors affecting changes in corporate equity size 11. Research on the introduction of cross-border capital by enterprises focuses on the effectiveness of equity governance effects 12, and few literatures focus on the advantageous strategies of Chinese enterprises in attracting cross-border capital 13.” --This paragraph contains the numbers 10, 11, 12, 13. Does these numbers correspond to citations in your reference list? If so, please put the numbers in brackets. Proceed so with all similar cases.**

Thanks for your helpful suggestion.

We have reviewed the full text. In the text, we put the citation numbers for the references in brackets.

**2.In the above mentioned paragraph, please replace “few literatures” with “few scholars” or “few manuscripts”.**

Thanks for your helpful suggestion.

We replace “few literatures” with “few scholars”.

**3.Additionally, ensure that all references listed appear as citations within the text. For example, I could not identify the in-text citation of the reference numbered [70] in your reference list.**

Thanks for your helpful suggestion.

In the text, “the reference numbered [70]” is in the fourth paragraph of “7.2 Further Discussion”.
